# Advances in Nanotechnology towards Development of Silver Nanoparticle-Based Wound-Healing Agents

**DOI:** 10.3390/ijms222011272

**Published:** 2021-10-19

**Authors:** Zimkhitha B. Nqakala, Nicole R. S. Sibuyi, Adewale O. Fadaka, Mervin Meyer, Martin O. Onani, Abram M. Madiehe

**Affiliations:** 1 Organometallics and Nanomaterials, Department of Chemical Sciences, University of the Western Cape, Bellville 7535, South Africa; 3340071@myuwc.ac.za; 2 Department of Science and Innovation (DSI)/Mintek Nanotechnology Innovation Centre (NIC)-Biolabels Node, Department of Biotechnology, University of the Western Cape, Bellville 7535, South Africa; nsibuyi@uwc.ac.za (N.R.S.S.); afadaka@uwc.ac.za (A.O.F.)

**Keywords:** antimicrobial agents, nanotechnology, silver-based therapy, silver nanoparticles, wound healing

## Abstract

Since antiquity, silver-based therapies have been used in wound healing, wound care and management of infections to provide adequate healing. These therapies are associated with certain limitations, such as toxicity, skin discolouration and bacterial resistance, which have limited their use. As a result, new and innovative wound therapies, or strategies to improve the existing therapies, are sought after. Silver nanoparticles (AgNPs) have shown the potential to circumvent the limitations associated with conventional silver-based therapies as described above. AgNPs are effective against a broad spectrum of microorganisms and are less toxic, effective at lower concentrations and produce no skin discolouration. Furthermore, AgNPs can be decorated or coupled with other healing-promoting materials to provide optimum healing. This review details the history and impact of silver-based therapies leading up to AgNPs and AgNP-based nanoformulations in wound healing. It also highlights the properties of AgNPs that aid in wound healing and that make them superior to conventional silver-based wound treatment therapies.

## 1. Introduction

Wound healing is a physiological process that occurs automatically as the skin or other body parts respond to an injury [1,2]. Although the process is natural, there are many formulations and therapies used to accelerate the wound-healing process. Among these are traditional medicines that involve the use of natural products, such as aloe vera, honey, cotton, wool, etc. Some of these products are developed or incorporated into conventional wound-healing therapies to improve healing rate and provide moisture to the wound site [3,4,5]. Conventional wound therapies include wound dressings, creams, ointments, bandages, growth factors, surgery, etc. Silver-based therapies have dominated this field for centuries and are still practiced to date. Despite the usefulness of these conventional therapies, including the silver-based therapies, they possess many limitations, such as high cost, skin discolouration, disruption of the nervous system and pneumothorax and incomplete healing [3,4,6,7]. As a result of the limitations associated with the current wound therapies, new and innovative strategies are necessary to develop therapies that will give effective and sustainable wound healing to improve quality of life for the patients. Most often, therapeutic strategies are improved by bringing in new technologies to enhance the properties of the existing technologies.

Nanotechnology provides an opportunity to develop novel wound-healing systems that will not only be effective but also economical. Nanotechnology is an interdisciplinary field that deals with the fabrication, assembly and utilisation of nanomaterials with a diameter of 1–100 nm [8,9,10]. Metallic nanoparticles (MNPs) produced from inorganic sources proved to be more effective when compared to organic nanomaterials due to their unique intrinsic physicochemical properties [3,11]. Their small size and high reactivity enable them to penetrate deeper into the wound site and afford better association with biological components. Due to their high surface-area-to-volume ratio, they can serve as a vehicle for other therapeutics, leading to accelerated and synergistic healing properties [7,12]. Moreover, MNPs have enhanced mechanical strength, can facilitate controlled drug release and have been shown to have toxic effects towards both fungi and bacteria, these properties [1] further confirm them as excellent candidates for various medical applications, including wound healing [13]. Among these, AgNPs are the most researched, highly utilised and commercialised nanomaterials for various biomedical purposes, especially for applications in wound care [5,11]. This stems from their excellent antimicrobial activity, where since antiquity, silver and its derivatives have been used to avert the occurrence of infections during wound healing [5,14]. Thus, this review covers the use of silver-based therapies, such as silver sulphadiazine (SSD) leading up to AgNP-based formulations in wound care and wound management. 

## 2. Pathophysiology, Economic and Health Burden of Wounds

The skin is the largest organ in the human body and covers approximately 2 m^2^ area of the entire body [15,16]. It serves as a protective barrier against infections to maintain the body’s homeostasis [16]. When the skin or any other organ is subjected to a heavy force [2,4], this causes damage to the epithelial integrity of the tissues, or to deeper subepithelial tissues, such as dermis, nerve, facia and muscle tissues [17]. The external stimuli that are capable of creating a wound include: surgical operations, cuts, chemicals, pressure, friction, heat, stress, shear and certain diseases [10,18]. 

Acute wounds should heal between 2–3 weeks at most, followed by the remodelling stage, which occurs over a period of up to two years. Normal healing is characterised by scab formation and little or no infection. Usually, the immune system fights off the infection when present. But in the event of serious microbial colonisation, the immune system cannot clear the infection and the wound progresses to the chronic stage [3] and fail to heal in a predicted time span and systematic manner [1,17,18,19]. Chronic wounds are more prone to bacterial infections and are difficult to maintain [1]. These wounds are characterised by an elevated degree of bacterial colonisation, increased inflammation, decreased oxygenation on the subepithelial tissues, damaged fibroblast and delayed re-epithelisation [3,7]. The most common chronic wounds include, but are not limited to, leg ulcers, fungating wounds, pressure ulcers, diabetic foot ulcers (DFUs), etc. [4,13,18]. 

Wounds pose a social challenge to patients and their families, as well as a huge economic burden on healthcare systems. Approximately 8.2 million people were affected by wounds in the US, as reported in a retrospective analysis of Medicare beneficiaries, in 2014. The combined treatment and management costs in the wound category varied between $28.1 billion and $96.8 billion. The management costs of DFUs were among the highest, at an estimate of $9–$13 billion [20,21]. These costs are expected to rise, as the number of diabetic people in the world has increased to 463 million since the time of the study, and is expected to reach 700 million by 2045 [21]. The probability of a diabetic person developing a DFU is between 15% to 25% if the diabetes is not properly managed. DFUs present a 14% to 24% chance of lower leg amputations (LLAs) as a result of peripheral neuropathy and unmanageable foot infections. LLAs are associated with a reduced quality of life, since amputation patients often struggle to adjust to social and demographic environments. Furthermore, LLAs are associated with a higher rate of mortality in diabetic patients; a recent study reported the 30-day postamputation mortality rate to be 9.8%. If the patient survives beyond the 30-day mark, they are at an increased risk of undergoing other amputations. Mortality rates at one and four years postamputation were reported to be 18% and 45% for minor LLAs and 33% and 65% for major LLAs, respectively. Depression has been recently identified as one of the risk factors contributing to high mortality rates after major LLA, among other factors, such as age, kidney disease and other diabetes-related complications [22].

### Current Wound Treatment Therapies and Their Limitations

**Figure 1:**
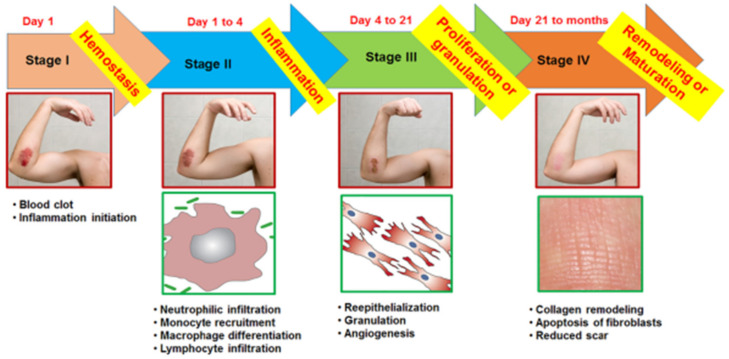
The basic stages of wound healing. Stage I is the homeostasis phase and it occurs immediately after injury. Stage II is inflammation, where a number of enzymes and growth factors are produced to fight off infection. Stage III represents the proliferation stage; this is where extracellular matrix (ECM) and collagen are produced, leading to re-epithelisation. The final stage, stage IV, is the remodelling stage, where wound closure occurs. Reprinted with permission from Elsevier [13].

Wound healing is a physiological process that is activated by the body in order to heal [23] and repair itself after injury or trauma [2,24,25]. It is an instantaneously initiated process that follows four well-orchestrated and sometimes overlapping stages as summarised in Figure 1; viz, homeostasis, inflammation, proliferation and remodelling [10,26,27]. Each stage is directed and controlled by a variety of factors, such as cytokines, chemokines, enzymes and growth factors [26,27,28]. 

Although wound healing occurs naturally, there are many engineered materials that are used to aid in the process and ensure that healing progress without interruption or failure. Accelerated wound closure, prevention of infections, complete healing and minimal scarring are the main goals of wound care and management [1,29]. In the past, a variety of natural products, such as plant extracts (aloe vera), honey, cotton and wool, have been used to facilitate wound healing [3]. Unfortunately, variations in treatment outcomes between patients, as well as the inability to provide sufficient moisture to the wound and allergic reactions were observed [7]. These factors limited their use and were replaced by other therapies, such as stem cell therapy, artificial and biomaterial wound dressing, bioengineered skin substitutes, vacuum-assisted wound closure, growth factor therapy, vascular surgery and silver-based therapy [2,3,4,25]. 

Stem cell therapy accelerates the rate of wound healing by enhancing the immune system and angiogenesis. It has the ability to reinstate the tissue to its preinjury form [30]. Artificial and biomaterial agents are integrated into wound dressing materials to mimic the physical and biological characteristics of neighbouring tissues and encourage wound healing [1,29]. Many bioengineered skin substitutes have been formulated to target dermal injury and restore dermal architecture [3]. 

Vacuum-assisted wound closure employs negative pressure therapy to speed up collagen production, maximise tensile strength and dermis irrigation. It promotes cell proliferation and angiogenesis, clears bacterial infection and drains lymph nodes. Furthermore, it can produce new granulation tissue after surgical debridement of infected wounds, making it useful in vascular surgery [3,4]. Since chronic wounds are identified by local exhaustion of growth factors, topical administration of growth factors, such as the Food and Drug Administration (FDA)-approved recombinant human platelet-derived growth factor (rhPDGF), have been employed in the wound-healing process [3]. These treatments achieve normal healing by activating various cells needed for the stages of the wound-healing process [3,7]. Vascular surgery is a very common strategy for treating and managing non-healing chronic wounds, including venous leg ulcers. The possibility of ulcers developing after surgery is very low, which makes this strategy highly attractive for the treatment of wounds [4].

Despite the potential of these conventional wound therapies, their clinical application is restricted due to various limitations. These include high costs, as in the case of vascular surgery, growth factors and stem cell therapies, while reduced moisture, low mechanical strength and decreased impregnation, among other issues, are associated with various wound dressings [6]. Moreover, decreased vascularisation and immune rejection are also associated with biomaterial wound dressings [7]. Other therapies can disrupt the central nervous system, such as hyperbaric oxygen therapy, which causes oxygen toxicity and pneumothorax [4]. Topical administration of growth factors requires a suitable dispensing vector to promote re-epithelialisation [3]. Excess wound exudates from non-healing chronic wounds also disturb stem-cell proliferation and cause growth factor therapy failure. Despite FDA approval, growth factor therapy fails to heal up to 70% of chronic ulcers [7]. As a result, new, innovative and economical strategies are urgently required to address these limitations and afford better wound healing to patients. Silver-based therapies present a cost-effective alternative to current therapies and have been extensively used in wound healing for centuries. They can be easily administered (topically) and can be effortlessly added into other wound dressings to afford synergistic effects [14,31].

## 3. Medical Properties of Silver

### 3.1. Brief Medical History of Silver

Silver is a naturally occurring metal positioned at 47 on the periodic table of elements, and has a characteristic pale and shiny appearance [14,32,33]. It is denoted as Ag, short for “argentums”, and belongs to the noble group of transition metals alongside gold, copper and platinum [5,33,34]. In its pure form, Ag possesses extrinsic properties, including high thermal and electrical conductivities, compared to other metals, while maintaining a very low contact resistance [14,33,34].

The antimicrobial properties of silver have been known since ancient times, and as a result this metal and its derivatives were widely used for various applications including, but not limited to, wound care, disinfection and storage of water [32,35,36]. The first reported use of silver was around 3500 BC in predynastic Egypt, where silver coins were used as a currency carved with the head of King Ptolemy X or Aegis eagle [14,35].

Silver containers were later used to transport and store water for the Persian kings who believed that the silver preserved the freshness and purity of the water [5,14]. In 335 BC, Aristotle advised Alexander the great to store his drinking water in silver jars, and to boil it before use when embarking on one of his many campaigns [35]. Throughout ancient civilisation, the Greeks and Romans stored water and other beverages, such as wine, in silver jars to avoid spoilage, which was useful during times of war as there was often no fresh water on the battlefields [36]. Furthermore, silver coins were used as disinfectants to sterilise and preserve liquids [30]. The Australian and American settlers would insert either silver coins or tableware into their drinking water and milk barrels to preserve the liquids [31,34,36]. As it was known that silver prevented decay and spoilage, nobles began using silver cutlery, and even to this day silverware is used for serving food, water and other drinks [34].

### 3.2. Silver Use in Medicine

The medicinal use of silver dates back to 1000 BC, when ancient civilisations used it as a prophylactic and as a treatment for various diseases [30]. According to The Merck Index First Edition published in 1889, silver nitrate (lapis infernalis) or lunar caustic was one of the silver salts listed for pharmaceutical purposes [34]. The first information about the medicinal use of silver nitrate for the treatment of wounds was reported in 702–705 A.D. [14,36]. 

In 980 A.D., silver filings were used to prevent heart palpitations and foul breath. It was also believed that silver could cure epilepsy, because when a man who was suffering from epilepsy accidentally swallowed a large silver coin that was placed in his mouth to stop him from biting his tongue during a seizure, it completely cured his epilepsy [36]. In the late 19th century, the very first scientific paper detailing the medicinal use of silver was published by F. Crédé, who used 1% silver nitrate solution as eye drops to cure blindness caused by postpartum eye infections in infants [37]. This therapy was highly effective in reducing the occurrence of ophthalmia neonatorium in newborns from 7.8% to 0.13% for a period of 13 years. As a result, this therapy was accepted in many countries across the world. Some even mandated it by law, and it continued until it was overtaken by the discovery and use of antibiotics [36]. F. Crédé’s son investigated the effect of metallic silver on *Staphylococci* and *Streptococci* in vitro. He found that when slivers of silver were placed in a dish that was then inoculated with microorganisms, no microorganisms would grow in the place that was occupied by the silver after 24 h [31]. Furthermore, Credé developed two silver salts, namely silver lactate and silver citrate, as means of delivering Ag^+^ ions during in vitro antibacterial studies [31,38]. The medical use of silver salts was not only limited to the treatment of bacterial infections caused by conditions such as gonorrhoea and syphilis, but also extended to the treatment of mental illnesses as well as nicotine addictions [5]. 

Silver also played a major role in battlefield surgery, where Ambrosie Pare, a royal surgeon, successfully used silver clips in facial reconstructions [14]. Another impressive medicinal use of metallic silver in the 19th century is attributed to Konrad Röntgen for his discovery of the X-ray. He realised that X-rays activated silver halide crystals, making it possible to record radiographic images [36]. Older editions of the British National Formulary also reported the use of silver nitrate in a body lotion [39], while other silver-based personal care products have also been in use since 1996, with no reports of toxicity or hypersensitivity [37]. By the beginning of the 20th century, numerous advances in the medicinal use of silver had already been reported, and the nature of the antimicrobial action of silver was better understood [31]. In this era, silver was mainly used in colloidal form for numerous applications, including germicides in hospitals. The efficacy of colloidal silver is described as bactericidal with no serious side effects [5]. Currently, silver and its derivatives are commonly used in the medical industry as coatings for catheters and implants, while its most important function is its use as a microbicide to prevent long term and recurrent infections in burns, diabetic ulcers and traumatic wounds [37].

### 3.3. Use of Silver in Wound Care

Silver is the most studied precious metal for use in combating bacterial infections [40]. The very first time silver was used in wound care was when the Macedonians used silver plates to treat and prevent surgical infections, and by doing so achieved better wound healing [36]. The Surgeons Mate, a textbook published in 1617, which described a solution containing 1:3 silver to nitric acid ratio used to treat leg ulcers, was one of the earliest publications to mention the wound-healing abilities of silver [31]. In 1895, Halstead successfully used silver wires to suture surgical wounds, and silver foil dressings to avoid postoperative infections [31]. Furthermore, during World War I, a silver leaf was placed onto the wounds of soldiers to prevent infections and to promote healing [5]. This was also commonly practiced in Ancient Mediterranean and Asiatic cultures [32]. 

The initial topical use of silver nitrate in wound treatment is attributed to Moyer and his co-workers, who used 0.5% of this silver compound to control the growth of *Pseudomonas aeruginosa* (*P*. *aeruginosa*) in severely burnt patients [37]. Grawits also reported on the antimicrobial properties of silver when he used a highly diluted silver nitrate solution to stop the growth of *Staphylococcus aureus* (*S*. *aureus*) [30]. Unfortunately, the bacteria later developed resistance against silver nitrate, prompting a change in formulation, which resulted in the introduction and application of SSD [37]. SSD is a manmade complex that was synthesised in the 1960s by complexing a silver ion (Ag^+^) to a sulphonamide antibiotic [34], and is still commercially available to date [39].

SSD was created in an attempt to retard the absorption of sulphadiazine, since it was observed that water-soluble compounds, such as sodium sulphadiazine, are quickly absorbed from wounds and excreted in urine [39]. As a result, the combination of silver and sulphadiazine achieved a synergistic effect against bacteria [35]. SSD was a far better alternative because it did not produce argyria, a serious side effect associated with silver nitrate-based products [37]. SSD was highly effective against a broad spectrum of bacteria because it slowly delivered the Ag^+^ ions to the wound site [14] compared to other silver compounds in the presence of sodium chloride, nutrient broth, DNA, human serum and bacteria. Other silver salts, for example silver nitrate, dissolved immediately and disappeared in the solution and reappeared as a silver chloride precipitate. Some other silver salts showed poor dissociation of Ag^+^ ions, releasing a very limited amount of silver over several hours. On the other hand, SSD released Ag^+^ ions in uniform concentrations at a controlled rate [35]. Hence, it rapidly became the gold standard for the treatment of burns, chronic ulcers and other skin wounds [5,35]. 

SSD has been used as one of the main ingredients, at 1% concentration, in many topically administered wound treatment agents, such as creams, ointments and dressings [5]. One of the most common SSD-containing creams, Flamazine™ (Smith & Nephew, Hull, UK), has been a mainstay topical treatment for infections pertaining to burns, acute and chronic wounds [41]. Another 1% SSD cream, Silvadene™ (Pfizer, New York, NY, USA), was successfully used to treat wounds in pigs, increasing epithelialisation by almost 30% [41]. Although SSD was proven to have antimicrobial activity against a broad spectrum of bacteria, some bacteria developed resistance to it. The bacterial resistance was attributed to the antibiotic component of the compound [37]. This led to the investigation of the combination of SSD with other compounds as topical wound therapies, such as Flammacerium, a combination of 1% SSD and cerium nitrate [42], and Silvazine^TM^, which is a mixture of 1% SSD and chlorhexidine digluconate [37]. 

When compared to Acticoat^TM^ (Smith & Nephew, Hull, UK) and Flamazine^TM^, Silvazine^TM^ was the more effective, killing Methicillin-resistant *Staphylococcus aureus* (MRSA), *P*. *aeruginosa*, *Escherichia coli* (*E*. *coli*), *S*. *aureus*, *Enterococcus faecalis* (*E*. *faecalis*), *Enterobacter cloacae* (*E*. *cloacae*), *Proteus mirabilis* (*P*. *mirabilis*) and *Acinetobacter baumannii* within 30 min. Acticoat^TM^ is a wound dressing that consists of a rayon–polyester core sandwiched in between two layers of high density polyethylene mesh coated with nanocrystalline silver [40]. Silvazine^TM^ killed all the bacteria within 30 min, while Acticoat^TM^ was only successful against *E*. *faecalis* and MRSA, with *P*. *mirabilis* and *E*. *cloacae* showing resistance to its activity [43]. Despite the limitations associated with the clinical use of SSD, such as the breakout of resistant strains, the impairment of wound healing and the development of systemic side effects [41], it is still listed as an essential medication by the World Health Organisation, and is highly recommended in the market for wound treatment [5]. 

A number of topically applied silver-based therapies have been extensively evaluated on chronic wounds with favourable outcomes [40,41]. As a result, a variety of silver-based dressings, including (but not limited to) Acticoat^TM^, Aquacel^TM^ Ag, Contreet^TM^, Actisorb^TM^ Silver 220 (Johnson and Johnson, New Brunswick, NJ, USA), Urgotul SSD^TM^ and Avance^TM^ have entered the market [40]. Table 1 highlights some of the silver-based dressings that are commercially available, as well as their advantages and disadvantages. In addition to bacterial resistance to silver treatment, a blue–grey discolouration of the skin (agyria) and other mucosal body parts often develops as a result of prolonged usage or contact with silver-based compounds [36]. Consequently, the silver-based systems still dominating the markets are currently being developed into new and innovative wound treatment technologies for the betterment of human health and well-being. Nanotechnology is an emerging branch of science that has the potential competency of solving the limitations of the bulk silver-based therapies at the molecular and submolecular levels using AgNPs [8,10,44].

## 4. Nanotechnology

Nanotechnology is an interdisciplinary field that deals with the production, manufacturing, characterisation and application of nanosized materials [8,10,44]. It deals with significantly smaller objects at the size range 1–100 nm [44]. There are generally two types, organic and inorganic nanomaterials. The organic nanomaterials include carbon-bearing particles such as liposomes, amongst others, while the inorganic nanomaterials consist of semiconducting nanoparticles, such as titanium dioxide and MNPs made from noble transition metals [12]. These nanomaterials have been incorporated into various nanosystems and nanodevices that are frequently used for research, as well as for commercial and clinical applications. This stems from their novel size-dependant properties, which make nanomaterials exceptional and essential for various aspects of human life [52]. Their small size equips them with distinct physicochemical and biological properties that are not observed in their bulk counterparts [53]. These unique features are attributed to quantum confinement effects and the proportionally larger surface areas that are not present in the macro compounds of the same composition [53,54]. As a result, their remarkable optical, electrical, magnetic, catalytic, biological and mechanical properties [55] make them excellent candidates for application in various fields, such as electronics, drug delivery, environmental remediation, biological labelling, chemical sensing and imaging, information storage, photonics and catalysis [56].

This review will mainly focus on the MNPs, specifically the AgNPs and their application in wound healing. Remarkable research in this field has already created the possibility of developing advanced therapeutic agents for wound healing [10].

### 4.1. AgNPs and Their Synthesis

AgNPs are without a doubt the most commercialised nanomaterials among the MNPs [11]. Based on the Nanotechnology Consumer Products Inventory, they appear in 24% of the registered products for various applications [5]. 

**Figure 2:**
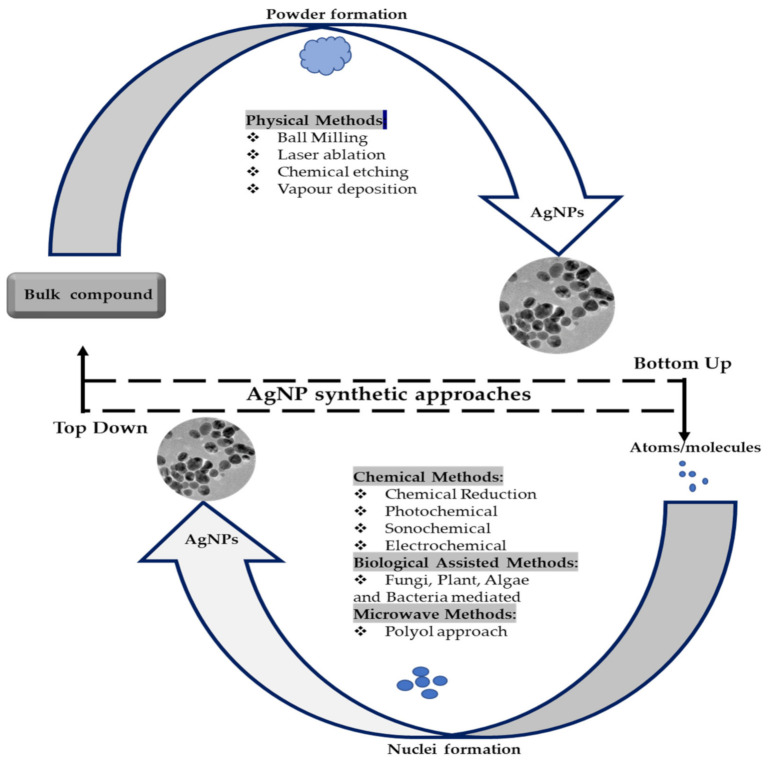
The bottom-up and top-down approaches for nanoparticle synthesis. Adapted from [57].

As with most nanomaterials, AgNP synthesis follows either a top-down or a bottom-up approach, as shown in Figure 2 [57]. The top-down approach involves the physical reduction of a bulk compound to its smaller constituents. In this method, a heavy force is used to crush the macromolecule into nanoparticles through physical and mechanical processes, such as UV irradiation, lithography, laser ablation, ultrasonic fields and photochemical reduction [5,58]. The physical reduction methods produce AgNPs with a narrow size distribution range and usually have fast processing times. However, they have many limitations, including the large space requirement for equipment, the long time required to reach thermal stability, the energy-intensive nature of the procedures, and they cannot be readily employed for scaling-up purposes [5,59]. 

In the bottom-up approach, small particles, such as atoms and molecules, are utilised as building blocks to obtain nanoparticles. Self-assembly or assisted assembly of these smaller particles results in the production of a complex nanoparticle [58]. The bottom-up approach is subdivided into the chemical, microwave and biological methods [60]. Chemical methods are widely used for the production of AgNPs in solution. They are prepared either in water or in other organic solvents [60]. Chemical methods are fast and efficient, however, these pose a major environmental concern due to the use of toxic chemicals as reducing agents and the production of toxic by-products [5,11]. To mitigate against these environmental effects, new alternative methods that are eco-friendly, cost effective and energy efficient are quickly emerging [53]. The development of green or biological synthesis has been extensively reviewed in literature [5,11,12,53,58,59]. 

Green synthesis works in exactly the same way as chemical reduction methods, however the capping/stabilising and reducing agents are replaced by environmentally benign materials, such as microbial (fungal and bacterial) enzymes and phytochemicals from plant extracts (leaves, roots, barks, flowers, fruits, peels and seeds). These biological entities produce nanoparticles that are biocompatible and are useful candidates for pharmaceutical and biomedical applications [61,62,63]. Although green synthesis is more favourable than chemical and physical methods, using microorganisms as reducing agents in nanoparticle synthesis is very complex, and requires numerous steps. The aforementioned problems resulted in the consideration of plant extracts for AgNP production, because they are abundantly available, easily extracted and do not require complicated procedures. Plant extracts are used for nanoparticle production mainly because they are highly accessible. The major challenge with plant-based synthesis is the production of AgNPs of uniform shapes and sizes [59,62,63,64], and the reduction mechanisms of biological methods are poorly understood [58].

#### 4.1.1. Properties and Bio-Medical Application of AgNPs

The physical and chemical properties of AgNPs, such as surface chemistry, shape, morphology, composition, coating/capping, size, surface charge and reactivity, amongst many others, are very important in their downstream applications in different fields [5,65]. These properties are notably important in their biomedical applications, such as antifungal [66], anticancer [67], antiviral [68], antiangiogenic [69], antileishmanial [70] anti-inflammatory [71] and antibacterial [72] activities. Due to the global increase in life threatening diseases and the emergence of multidrug resistance in pathogens against antibiotics [73], multiple medical applications have been demonstrated for AgNPs, such as sensors for disease diagnosis [74], nanocarriers for drug delivery [75,76], sensitisers in radiation therapy [77], electro spin resonance dosimetry, etc. [78]. This review focuses on the wound-healing properties of AgNPs, as discussed below. 

AgNPs have the potential to circumvent the previously mentioned limitations of silver-based compounds, such as bacterial resistance and skin discolouration, and are effective at very low concentrations. Some of the essential properties that make AgNPs highly useful in the treatment and management of wound healing, include their ease of synthesis by a variety of methods, and physicochemical properties, such as size and morphology, which can be easily manipulated for specific applications. AgNPs have antimicrobial activity against a broad spectrum of microbes, including drug-resistant types. Surface modification is easily achieved with AgNPs, which is extremely useful for targeted drug delivery and drug loading. AgNPs also have anti-inflammatory properties that aid in tissue regeneration. When combined with other drugs, AgNPs can afford a synergistic effect which leads to enhanced activities and reduced side effects. Lastly, AgNPs can be easily integrated into wound dressings materials for quick and sustainable effects [50].

#### 4.1.2. Properties of AgNPs That Aid in Wound Healing

**Figure 3:**
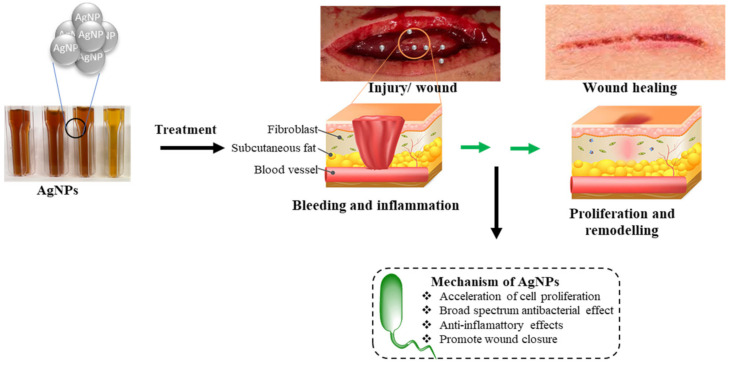
AgNP wound-healing mechanism. Exposure to AgNPs promotes wound closure by preventing bacterial colonisation and inflammation in the wound site. Open wound image adapted from [79].

AgNPs are used as therapeutic agents for wound-healing purposes mainly because of their remarkable anti-inflammatory and antibacterial properties [29], as depicted in Figure 3. When AgNPs come into contact with the affected area, they initiate neutrophil apoptosis by reducing the mitochondrial membrane potential, which then lowers the cytokine production. Consequently, this modulates or reduces inflammatory response, resulting in faster healing [25,64].

##### Anti-Inflammatory Properties of AgNPs

When foreign particles invade the body, the affected tissues trigger an early immunological response i.e., inflammation, to fight off these disturbances by producing a large number of pro-inflammatory cytokines, resulting in the activation of the immune system and the release of chemotactic and prostaglandin species. The released factors include the complement factors, interleukin (IL)-1, tumour necrosis factor (TNF)-α, and transforming growth factor (TGF)-β. AgNPs are biocompatible and are able to escape this involuntary inflammatory action, hence they are not neutralised by the immune system and can be used as anti-inflammatory agents [65]. AgNPs has exhibited excellent anti-inflammatory activities in animal models as well as in clinical trials by reducing the expression of pro-inflammatory cytokines, such as the transformation of growth factor-α and TNF-α [5]. Due to the anti-inflammatory properties of AgNPs, the topical application of AgNPs in the wound area reduces the release of cytokines and lymphocytes, and also reduces mast cell infiltration, which then promotes wound healing with minimal scarring [2,3,80]. Similarly, in diabetic wounds, AgNPs accelerated the rate of wound healing by activating the proliferation and migration of keratinocytes. Moreover, they aided in the differentiation of fibroblasts into myofibroblasts, which accordingly promoted wound contraction and speeded up the healing of diabetic ulcers [13].

The anti-inflammatory effects of AgNPs were also compared to the commercially available anti-inflammatory drug, indomethacin. Rats were treated for four hours with two doses (0.25 and 0.50 mL) of 250 ppm AgNPs, which revealed a decrease in inflammation in the rat bow oedema model in a dose-dependent manner. Furthermore, the AgNPs showed similar activity to indomethacin (reference drug) in reducing the rat bow oedema [71], indicating the potency of the AgNPs. In an in vitro study, AgNPs significantly reduced inflammation in dermal fibroblast and human keratinocytes by lowering the cytokine levels, and also reduced oxidative stress and promoted wound closure [26]. Topical application of AgNPs in mice with burn wounds showed decreased counts of neutrophils and low levels of IL-6, accompanied by an increase in the levels of IL-10, TGF-β, vascular endothelial growth factor (VEGF), and interferon (IFN)-γ. The AgNP-treated group healed faster (~27 days) compared to the SSD-treated (~37 days) and untreated (~35 days) groups. Furthermore, the AgNP-treated wounds resembled normal skin and showed normal hair growth as well as reduced hypertrophic scarring [81]. As a result of their anti-inflammatory activity and ability to promote cell growth, AgNPs are useful tools in wound healing [80]. They are responsible for the differentiation of fibroblasts into myofibroblasts, which encourages wound contraction, faster healing, and quickens the proliferation and relocation of keratinocytes [82]. The effects of AgNPs on keratinocytes and fibroblasts of rodents were studied in an excisional wound model. The histological and ex vivo tests showed that AgNPs enhanced proliferation and movement of keratinocytes, and stimulated the differentiation and maturation of keratinocytes, and hence prompted wound contraction. The group of mice treated with AgNPs showed better wound closure than the mice treated with SSD [83].

##### Antibacterial Properties of AgNPs

One of the major issues associated with wound closure is colonisation of the wound by infectious pathogens [84]. Although bacteria in the human microbiota are helpful in stopping other infectious pathogens from colonising the wound, they too can be harmful and can impair wound healing if they reach a dangerous threshold at the wound site. *S. aureus* and *MRSA* are often found in the beginning stages of wound healing, while *P. aeruginosa* and *E. coli* are observable in the chronic stages, as they infect deep skin layers [3]. AgNPs are used in wound care management to avoid secondary infections, as they are active against various strains that can delay the normal healing process [80,85].

Compared to silver salts and complexes, AgNPs exhibit higher antibacterial efficacy [5] against an astonishing number of pathogenic Gram-positive and Gram-negative bacteria [86]. This activity is influenced by several AgNP parameters, most especially their size, morphology, concentration, surface charge and coating. The size and charge of AgNPs allows them access to microorganisms [14,34,73]. Many multiresistant strains succumbed to the AgNPs [5]. The antibacterial mechanism of AgNPs is highly complicated and poorly understood [53]. Nevertheless, the antibacterial action is initiated by the binding of AgNPs to the cell membrane and penetrating the cytoplasm of the bacterial cell where they interact with proteins, DNA and enzymes that contain phosphorus or thiol groups [3]. Once the AgNPs are inside the bacterial cell, the oxidation of AgNPs in aqueous medium triggers the release of Ag^+^ ions under acidic conditions. The Ag^+^ ions are believed to be the agents that carry out the bactericidal effect. This action might not be entirely through the AgNPs by themselves [1]. The AgNPs also attack and destroy the respiratory chain, resulting in bacterial cell death [2].

The antibacterial activity of AgNPs against *E. coli* was reported in previous studies, which showed that the AgNPs formed holes in the bacterial cell wall and accumulated in the membrane, resulting in bacterial death [87]. *S. aureus*, *MRSA*, *P. aeruginosa* and *E. coli* were all reported to be susceptible under *in vitro* conditions to chemically fabricated 8–50 nm AgNPs [88]. In another study, 26 nm green-synthesised AgNPs exhibited impressive antibacterial activity against *S. aureus*, *E. coli* and *P. aeruginosa* [70]. The antimicrobial efficacy of AgNPs aids in the removal of the pathogenic microbes that might disturb and impair the normal stages of wound healing [2].

#### 4.1.3. Nanoformulations of AgNPs in Wound Healing

**Figure 4:**
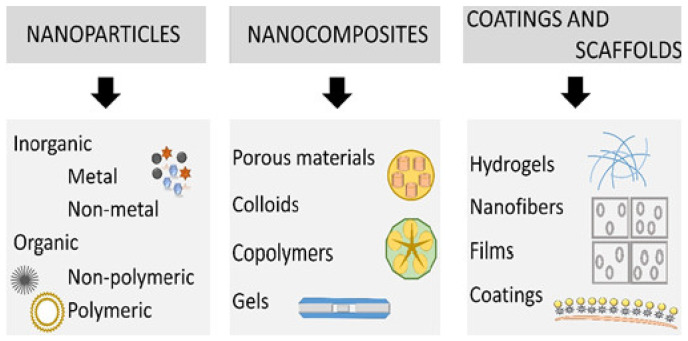
Main types of nanomaterials explored in wound treatment. Reprinted with permission from MDPI [3].

AgNPs have demonstrated potential wound-healing abilities, but when combined with other wound dressing materials, they were highly effective in killing microbes that may cause infections at the wound site [2]. Furthermore, owing to their small size, they have a very high surface-area-to-volume ratio which enables surface functionalisation with biomolecules. Beneficial biomolecules, such as DNA and drugs, can be incorporated to enhance their activity and prolong their circulation time [44]. As a result of the aforementioned properties together with their inherent broad-spectrum antimicrobial activity, AgNPs are highly utilised as topical agents in wound-healing applications in combination with other materials [70]. The main categories of the nanohybrids employed in wound treatment are depicted in Figure 4 [3].

In wound therapy, nanomaterials can be used in two ways. Nanomaterials that show distinctive characteristics can be instrumental in wound treatment, while nanomaterials in general can be used as delivery channels for therapeutic agents [3,7]. Nanomaterials with intrinsic wound-healing properties in various stages of the wound-healing process include various types of MNPs, such as AgNPs, metal oxide, non-metal and metalloid based nanoparticles; while nanomaterials used in drug delivery include organic nanoparticles that have therapeutic benefits [4]. As previously mentioned, AgNPs are easily functionalised with different functional groups, and easily incorporated into various materials to fabricate nanocomposites and scaffolds, which afford them a synergistic activity in the treatments of wounds [3,87]. The most common nanocomposites and scaffolds impregnated with AgNPs are briefly discussed below.

##### AgNP-Based Nanocomposites

Colloids, gels, porous and polymeric materials embedded with AgNPs all belong to the subclass of nanocomposites. These materials are enriched with many useful groups, such as amino acids, proteins, phenols and alkaloids, which are used for the reduction and stabilisation of AgNPs. The nanoparticles and the phytochemicals present in these materials work in synergy to speed up the wound-healing process [3]. For example, *Catharanthus roseus* leaf extract which is a medicinal plant that has a high content of alkaloids, was used as both the reducing and stabilising agents in the synthesis of AgNPs. The AgNPs demonstrated remarkable wound-healing properties and reduced the wound size over 4 days in Wistar Albino rats when compared to the untreated controls and rats treated with the *Catharanthus roseus* leaf extract. The wounds exposed to the AgNPs also showed no signs of pus formation, bleeding or contamination, whereas the control wounds showed signs of inflammation during the treatments. Furthermore, at the end of the experiment the alkaloid-enriched AgNPs achieved 98% wound closure, while the control wounds achieved only 85%. It was then suggested that the remarkable wound closure and reduced wound size could be due to the antibacterial effects of the AgNPs which prevented wound infection and reinstated the integrity of the tissues [89]. In another study, alkylated ε-Polylysine (EPL)-*g*-butyl@AgNPs were prepared *in-situ* and evaluated *in vitro*. The nanocomposite exhibited polyvalent and synergistic effects against *P. aeruginosa* and *S. aureus* without any resistance to the treatment. Furthermore, EPL-*g*-butyl@AgNPs showed faster wound healing in a diabetic rat model [90]. Similarly, synergistic effects were reported when the AgNPs were formulated into polymers, gels, films, scaffold, etc., as described below.

(a) Copolymers

Chitosan is a naturally occurring biocopolymer consisting of glucosamine and N-acetylglucosamine [91]. It is derived from chitin, which is found within the skeletal systems of shrimp, crustaceans, yeast and fungi [92,93]. It is nontoxic, biodegradable, economical, biocompatible and able to retain moisture [94]. Furthermore, it is also capable of achieving haemostasis, preventing scars as well as speeding up the wound-healing process [91,93]. It has therefore been used widely as a wound-healing biomaterial in different forms, such as hydrogels [95], membranes [96], scaffolds [97] and sponges [94]. There are numerous chitosan-based dressings available in the market; for example, Syvek-Patch^R^, Chitopack C^R^, Tegasorb^R^, HemCon Bandage™ and KytoCel^R^ [94]. Although it exhibits remarkable wound-healing abilities, the use of chitosan in infectious wounds is limited because it does not exhibit any significant antibacterial activity [91,92]. To overcome this, antibacterial agents, such as AgNPs, have been incorporated onto chitosan dressings. For example, Oryan and colleagues used chitosan as a stabilising agent in a one-pot synthesis of AgNPs. The chitosan enhanced the biocompatibility of the AgNPs and achieved superior antibacterial action against various strains. Also, in vivo tests revealed that the chitosan–AgNPs decreased inflammation and improved granulation tissue formation and re-epithelialisation [92].

Another highly utilised natural polymer in wound healing is gelatin, a protein derived from the direct hydrolysis of collagen. Much like chitosan, it is nontoxic, biocompatible and biodegradable [98]. Chemical modification and drug loading are easily achievable with gelatin, due to the presence of many beneficial functional groups in the protein’s structure. Additionally, the effective control of drug release and target delivery, as well as drug stability, are some of the many advantages of encapsulating drugs in gelatin [99]. Curcumin was successfully encapsulated into gelatin-stabilised AgNPs (GelCurAg) via a two-step fabrication process. In vitro studies showed that the GelCurAg nanocomposite could be a prospective biocompatible material for the treatment of infected wounds, as a result of its promising antibacterial and antioxidant activities [99].

(b) Gels

Nanogels are highly utilised in wound healing because they have many useful properties, such as swelling, a hydrophilic nature, softness, biocompatibility and stimuli–responsive behaviour. Furthermore, drug encapsulation is easily achievable with nanogels because of their 3D structure, which also protects the loaded drugs from in vivo elimination and degradation, resulting in a controlled and targeted drug delivery [25]. Wu et.al produced bacterial cellulose (BC) gel membranes impregnated with AgNPs (AgNP–BC) for antibacterial wound dressing. The AgNP–BC were evaluated in vitro and in vivo with compelling results [100]. The antibacterial activity of AgNP–BC against *S. aureus* was higher than the positive control (a commercially available gauze) with 3.46 mm and 2.03 mm zones of inhibition, respectively. Furthermore, BC on its own had no activity against the bacteria, indicating that it is solely AgNPs that are responsible for the antibacterial behaviour of the AgNP–BC. The AgNP–BC exhibited great wound-healing capabilities in vivo in a second-degree wound rat model, by regenerating new epidermal and dermis thickness. The AgNP–BC effectively reduced bacterial contamination on the wound site and provided better local surroundings for scald-wound healing [100].

In another study, *Ocimum sanctum* leaf extract, which contains phytochemicals, such as ursolic acid, rosmarinic acid, oleonic acid, orientin, apigenin, luteolin, moludistin, carvacrol, eugenol and caryophyllene, was used as both the reducing agent and the stabilising agent in the synthesis of AgNPs. Moreover, the phytochemical-enriched AgNPs were loaded into a Carbopol nanogel that was then evaluated for antibacterial and wound healing activities. In vitro tests showed that the AgNP-infused nanogels developed a zone of inhibition, of approximately the same diameter as that of a commercially available silver nitrate gel, against all tested pathogenic species. Also, the fabricated AgNP gel stimulated faster healing in partial thickness burns compared to both the negative and positive controls. The wound closure time was shorter and the wound size was significantly smaller, reducing by 96.20% within the 14 day period of the study [101]. Although nanogels check all the boxes for an ideal wound-healing formulation, they have some setbacks associated with the dynamics of their pharmacology, metabolism and kinetics. More research is needed to resolve these issues before nanogels can be successfully used in clinical trials [25].

(c) Porous materials

As previously mentioned, an ideal wound-treatment formulation should sustainably provide sufficient moisture to the wound site for better and quicker healing. Apart from that, it should also be able to soak up extra and unwanted exudates generated by the wound [3]. Researchers have tirelessly investigated hydrogels, films and foams for this purpose [96]. In one study, a highly porous material formulation was produced using BC. BC is a nanofibrous 3D network that has an optimal water retention capacity [100]. It is praised as a natural wound dressing, however it lacks antimicrobial activity. Thus, AgNPs are often incorporated into their hydrophilic structures to prevent secondary wound infections [85]. The AgNP-functionalised BC (AgNP/BC) membranes showed advanced bacterial death when compared to unloaded BC membranes. The AgNP/BC membranes were suitable for wound-healing applications because of their low toxicity, since Ag^+^ ions were released at a controlled rate, indicating the high stability of the AgNPs in the matrix membrane. AgNP/BC membranes are highly stable in moist surroundings, which is a requirement for an ideal wound-healing formulation. They can be stored for longer periods of time after their synthesis, and can be used in general wound healing and/or surgical cases [85].

AgNP-loaded konjac glucomannan (KGM) composite sponges were produced via a two-step fabrication process [16]. KGM is a highly water-soluble polysaccharide packed with carbonyl and hydroxyl groups through which the molecule can attract numerous water molecules via H-bonding and van der Waals forces, equipping it with superior water retention properties [16,29]. Moreover, it is also nontoxic, biodegradable and biocompatible, and therefore suitable for wound-healing purposes [16]. KGM/AgNP composite sponges exhibited longer water retention, superior mechanical activity and better antibacterial activity against *E. coli* and *S. aureus* compared to pure KGM sponges. Their good water-absorption properties enabled wound exudate absorption and kept the wound site clean. Moreover, their water retention led to tissue regeneration and pain relief. In addition, KGM/AgNPs showed shortened healing time in rabbit skin wounds, and thus a potential dressing material for wound-healing applications [16].

##### Coatings and Scaffolds

Scaffolds, such as hydrogels, nanofibres and films, have the ability to imitate the properties of the ECM, and are highly utilised in formulations for wound healing [29]. ECMs are fibrous in nature and possess nanoscale features that are attractive for wound-healing purposes, which is why researchers are using materials such as polymers to mimic these structures [7]. Often, AgNPs are integrated into scaffolds in an attempt to accelerate the wound-healing process [80]. Numerous methods, such as electrospinning, phase separation and self-assembly, are employed in nanotechnology for the fabrication of scaffolds [3].

(a) Nanofibres

Electrospinning is the most commonly used fabrication technique for the production [25] of uniform and highly porous polymeric nanofibrous scaffolds that exhibit structural and physical properties analogous to that of ECM [7]. This makes them attractive as hybrid scaffolds for the attachment and formation of fibroblast cells in wounds [3]. Nanofibrous scaffolds are advantageous as a result of their large surface area and small pores, which have a high degree of porosity [29]. Poly(dopamine methacrylamide-co-methyl methacrylate) (MADO) is a mussel-inspired copolymer that was electrospun into a fibrous scaffold followed by surface modification with AgNPs. In vitro studies suggested that the MADO–AgNPs scaffold had high antibacterial activity against *E. coli*, *S. aureus* and *P. aeruginosa*. The scaffold was nontoxic to mammalian cells. Wounded mice treated with MADO–AgNPs had no visible wounds, visible redness, and minimal scarring after the 15th day [102]. In another study, electrospun collagen nanofibres loaded with AgNPs exhibited a slow release of Ag^+^ ions following pseudo-zero order kinetics. As collagen is one of the major structural components of ECM in the connective tissues, it was able to guide cell adhesion and differentiation. This then revealed the potential of this combination for wound-healing applications. Lastly, in vivo studies showed faster wound healing for AgNP-loaded collagen nanofibres compared to unloaded nanofibres [103].

A comparative in vitro and in vivo study between SSD and AgNP-loaded porous polycaprolactone (PCL)/polyvinyl alcohol (PVA) nanofibres showed that while both SSD and AgNPs exhibited superior and equal antibacterial activity against *S. aureus*, the SSD-loaded nanofibres showed higher toxicity towards fibroblast cells over 7 days. The full thickness wounds created on the Wistar albino rats healed faster with the AgNP-loaded nanofibres compared to the SSD-loaded nanofibres. Furthermore, accelerated angiogenesis, re-epithelialisation and remodelling were observed for the AgNP-loaded PCL/PVA nanofibres [104]. A successful production of covalently crosslinked alginate fibres loaded with AgNPs was also reported for wound-healing purposes. The alginate fibres provided a strong platform for the delivery of AgNPs to the wound site, and thus decreased inflammation and increased epidermal thickness, resulting in accelerated wound healing [105].

(b) Hydrogels

Hydrogels are made up of a network of polymers that have a vast number of hydrophilic groups with cross-interactions that form a 3-dimensional matrix capable of trapping liquids, such as water or wound exudates [3]. As a result, they can rehydrate necrotic skin tissues and advance autolytic debridement [29]. Not only do they provide the wound with sufficient moisture, they also enable gas diffusion and absorb wound exudates, thereby preventing secondary infections [3]. Zwitterionic hydrogels (ZWDO) have attracted a lot of attention as potential wound-dressing materials because of their superhydrophilic nature, bacterial and protein adsorption and because they do not attach to skin cells. AgNPs integrated into antifouling ZWDO inhibited bacterial growth and killed *E. coli*, *S. aureus* and *P. aeruginosa*. Furthermore, ZWDO–AgNPs showed a 98% decrease in wound size and closure by day 15. Due to the fact that they do not attach to skin, they can be easily removed without causing pain and damage to the newly generated skin tissues during the periodic removal and replacement of wound dressings [102]. In another study, thermosensitive and injectable hydrogels derived from hyaluronic acid (HA), corn silk extract (CSE), and AgNPs exhibited high antimicrobial activity against various Gram-negative and Gram-positive strains. The thermosensitive hydrogels promoted wound closure and regeneration [106]. Gelatin–PEG–dopamine hydrogels loaded with AgNPs exhibited high antibacterial activity against *E. coli* and *B. subtilus*, as well as excellent biocompatibility and low cytotoxicity, qualifying them as potential wound dressings [107]. Various gelling agents, such as sodium carboxymethyl cellulose (Na CMC), sodium alginate (Na alginate), hydroxypropylmethyl cellulose, Pluronic F-127, and chitosan have all been used to prepare AgNP-loaded hydrogels [95]. The effect of the gelling agent on the spreadability, viscosity, drug release and antibacterial activity of the hydrogels was investigated in vitro and in vivo. All the formulations exhibited antibacterial activity against both Gram-negative and Gram-positive bacteria. In vivo studies indicated that Na CMC hydrogels loaded with AgNPs provided greater antibacterial and wound-healing abilities along with skin tissue regeneration and hair growth, when compared to Dermazin [95].

(c) Films

Using films from either natural or synthetic polymers, such as alginate or PVA, as wound dressings can enhance the resistance, bioadhesiveness and flexibility of the dressing. However, the fabrication procedure should be carefully selected, as films produced via film casting tend to be dense, have low gas and vapour permeability [29]. *Bombyx mori* cocoons possess a protective ability that is analogous to the way the skin protects the human body, hence the silkworm cocoon was evaluated for wound-healing activity. A novel silkworm cocoon-based wound-film (SCWF) dressing loaded with AgNPs was successfully prepared and evaluated for biocompatibility and antibacterial activity. Moreover, in vivo experiments in white rabbits indicated that the SCWF–AgNP dressing rapidly accelerated infected wound healing and demonstrated successful regeneration of intact and thick epidermis on day 14 [84]. PVA-coated nanofilms loaded with AgNPs demonstrated superior antibacterial activity against various strains at 100-fold loading, and the release rates of AgNPs that were lower than common dressings. When the nanofilms were deposited into the moist wound, the PVA dissolved, exposing AgNP-loaded nanofilm on the wound site, which then slowly delivered Ag+ ions, which killed the bacteria. These silver nanofilms displayed normal and complete wound closure through re-epithelisation [108]. Lastly, film and ointment forms of cellulose nanocrystals (CNCs) derived from *Dendrocalamus hamiltonii* and *Bambusa bambos* leaves and impregnated with AgNPs exhibited high water-absorption capacity and strong antibacterial activity, resulting in synergistic wound-healing effects. Histological examination with the nanocomposites demonstrated low inflammation and faster vasculogenesis during day 3 of the experiment, and on day 8, increased fibroblast and collagen contents were observed, whereas by day 14, accelerated neo-epithelisation was visible [109].

(d) Coatings

AgNPs are usually coated with various coating materials, such as polymers, surfactants, polysaccharides and carboxylic acid, to prevent aggregation and enhance their stability. Previously, AgNPs coated with PEG 6000, sodium dodecyl sulfate (SDS), and β-cyclodextrin (β-CD), showed stability and high antibacterial activity. The PEG-coated AgNPs showed enhanced stability, as evidenced by a lowered particle aggregation rate in solution [95]. The PEG-coated AgNPs were loaded into a Na CMC hydrogel, and this accelerated wound healing when compared to a commercial wound cream [95].

Nanoparticles are not the only materials that can be coated for wound-healing applications. Baygar demonstrated the use of both propolis and AgNPs to coat silk suture materials. The advanced wound-healing abilities of propolis, together with the antibacterial activity of AgNPs, worked in synergy to prevent surgical site infections and thus speed up wound healing [110]. Furthermore, medical implants are used more than ever before, due to the global increase of life expectancy and improvements in medical technology. However, these bodily implants can be a breeding ground for pathogens, resulting in bacterial infections. Thus, coating them with AgNPs can prevent these infections. A new coating material using PGLA fibrous membranes loaded with AgNPs demonstrated great biocompatibility and controlled the release of AgNPs, resulting in excellent antibacterial activity against both *S. aureus* and *E. coli* [111].

#### 4.1.4. Toxicity of AgNPs

Despite the global increase in manufacturing and commercialisation of nanotechnology-based products, there are still unresolved issues, such as the hazards of exposure to nanomaterials and their long- and short-term toxicity towards animals, humans and the ecosystem [86]. As previously mentioned, prolonged contact with conventional silver products causes argyria. Therefore, it is crucial to study the toxicity effects of silver at the nanoscale, since at this dimension the inert metal bursts with energy due to confinement effects. AgNPs pose a more harmful threat compared to their bulk counterparts because of their significantly smaller size and larger surface-area-to-volume ratios, which give rise to enhanced chemical and physiological performances [112]. For this reason, the interaction of this nanometal with humans and the environment needs to be explicitly investigated, so that unfavourable consequences can be known without ambiguity to ensure that proper steps can be taken to overcome the implicated health risks and potential cytotoxicity in cases of accidental and prolonged exposure [113].

**Figure 5:**
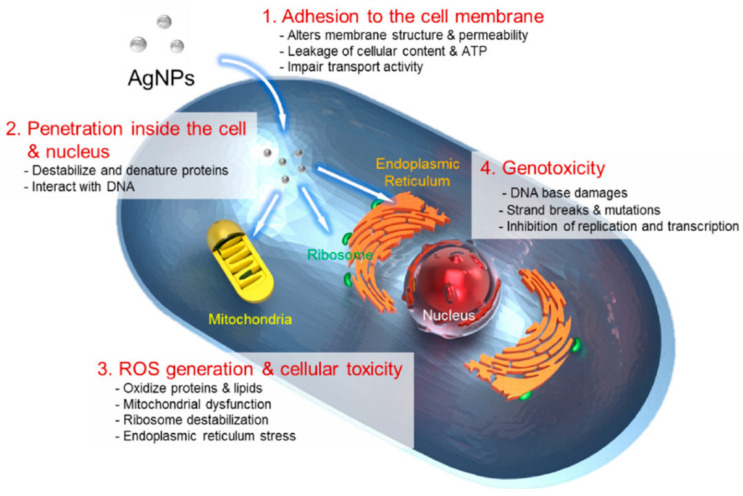
Routes of cytotoxicity action for AgNPs. (**1**) Adhesion to cell wall; (**2**) Cellular internalisation; (**3**) ROS generation; (**4**) Genotoxicity. Reprinted with permission from MDPI [61].

AgNPs may be administered orally, absorbed by the skin through direct contact or inhaled [114,115], the latter being the most common route of exposure among employees who fabricate and deal with nanomaterials [116]. Once they enter the body, they can travel and become toxic to many other organs [115,117]. If AgNPs contact the skin, they can be internalised by macrophages and cause inflammation, pronounced neovascularisation and unusual generation of ECM. Ingestion is another route by which they may enter the body, and they can thereby accumulate in the liver, since it is the main metabolic organ. This may cause liver dysfunction by inducing reactive oxygen species (ROS) and lowering the mitochondrial function. When inhaled, AgNPs may attack the respiratory system through oxidative stress [114]. There are several proposed mechanisms for AgNP toxicity; four of them are summarised in Figure 5. AgNPs are suspected to induce cellular toxicity by: (i) adherence of AgNPs to the cell membrane, resulting in physical destruction and cell membrane impairment; (ii) internalisation of AgNPs, which can cause malfunction of the intracellular organelle (mitochondria, vacuoles and ribosomes), and biomolecules (proteins, enzymes, lipids and DNA); (iii) induction of cytotoxicity and oxidative stress by generation of ROS and free radicals, which also disrupts the activity of biomolecules and intracellular micro organelles; and (iv) modulation of intracellular signal pathways [5,61].

The release of Ag^+^ ions inside the cell has been identified as the cause of AgNP cytotoxicity. However, this does not explain why AgNPs have higher cytotoxicity than other silver-bearing compounds [5]. Many scientists proposed that this toxicity is a result of the highly exposed surface areas and surface energies of the nanoparticles that allow for improved interaction with the cells. The size of AgNPs also plays a crucial role, and in most cases, smaller sizes have higher toxicity effects compared to larger sizes [112]. However, these toxicity effects depend on the study areas for different groups. Numerous papers reported that AgNPs are less toxic towards animal cells compared to bacterial cells, creating a curative opening that can kill bacteria without posing any threat to animal cells [118]. This stems from the highly complex structure and properties of AgNPs that are not present in the bulk material, so that higher Ag^+^ ion concentrations are needed to exert the same toxic effect on animal cells [117]. It is for this reason that the half-maximal inhibitory concentrations (IC_50_) of AgNP that kill bacteria pose no cytotoxic effect on animal cells [119]. AgNP/chitosan dressings enhanced cell growth on both human embryonic kidney (HEK293) and hepatocyte (L02) cells, while they imposed a toxic effect on *S. aureus*, *E. coli* and *MRSA* [94].

## 5. Future Perspectives

Nanotechnology has brought many exciting avenues to various fields above and beyond medicine. Among MNPs, AgNPs are the most commercialised, and are available in many consumer products, such as sunscreens [120], toothpaste [121] and lotions. Although AgNPs are well known for their antibacterial activity [62], research over the years has shown that these nanomaterials have other health-related beneficial properties. They are used in medical applications as diagnostic [74], drug delivery [75,76], sensitising and therapeutic agents. The global rise of the health, social and economic burden of wounds is a growing concern that necessitates novel strategies to produce sustainable solutions. The commercialisation of wound-care materials is also expected to rise; indeed, it has been estimated that the global antimicrobial dressing sector will increase by 7.2% (1.8 billion dollars) between 2020–2027 [21]. Following on from the decades of success stories of metal-based therapies such as cisplatin, AgNP-based products certainly have much to offer, and show potential for the development of effective wound dressings, as highlighted in this review. This has led to increased fabrication and commercialisation of AgNP-based materials. As it stands, there are already 35 AgNP-based products for oral and topical use approved by FDA for clinical trials. Furthermore, because consumer products (packaging, clothing, disinfectants, dietary supplements, dressings) are not as highly regulated, there could be even more AgNP-based products already in the market. Although safer and greener methods for producing AgNPs with reduced toxic effects have been reported, there could be potentially a higher risk of exposure when AgNPs are produced in larger quantities. These products are most likely to be orally ingested or absorbed by skin, and additionally, they are non-biodegradable. These factors raise many concerns for human health and the environment [122]. As already explained, AgNPs can travel through the body, accumulate in various organs and cause serious harm. New fabrication methods are continuously devised to help minimise the bystander effects of AgNPs [62,63,64]. Disposal strategies need to be explored to minimise their exposure to the environment. The exciting properties shown by green-synthesised AgNP-based systems proves that cheaper and user-friendly treatments can be developed to reduce the current health-related economic burden. These may be especially useful in settings where resources are low. However, a main challenge faced by green AgNP synthesis methods and by implication the formulation of AgNP-based wound treatments, is the development of reproducible manufacturing protocols. Variations in the phytochemicals present within plant extracts for example can significantly affect the consistency in the synthesis of the nanomaterials. Another concern is the full characterisation of the composition of green AgNP synthesis, i.e. identifying all the phytochemicals within the AgNPs is a complex task. While these green synthesis methods supposedly produce nanomaterials that are more bio-friendly, it is of utmost importance to investigate the impacts and possible toxic effects of AgNP-based systems on the environment as well as on humans. These problems will have to be addressed before these treatments can pass regulatory processes.

## 6. Conclusions

Silver and its derivatives have been extensively used for decades to aid wound healing due to their antibacterial action. However, conventional formulations had several shortcomings, such as toxicity and bacterial resistance. AgNPs have novel properties attributable to their nanoscale size, and have now entered the fray to circumvent the shortcomings of conventional silver-based therapies. In addition to their wound-healing properties, AgNPs have anti-inflammatory and antibacterial properties that make them superior to conventional silver-based therapies in wound-healing applications. Furthermore, AgNPs can be integrated into a variety of other materials that have wound-healing activities to give rise to potent formulations, such as nanocomposites, coatings and scaffolds, to achieve synergistic effects that result in optimum wound-healing processes. The studies under review highlighted the feasibility of developing AgNP-based formulations that are not only economical, but have exceptional biological activities that are pivotal for wound healing. AgNPs are already used in various consumer products, and can be developed into various wound-care agents to prevent bacterial infections and accelerate wound closure.

## Figures and Tables

**Table 1:** Advantages and disadvantages of the commercial silver-based wound dressings.

Product Name and Manufacturer	Formulation	Advantages	Disadvantages	Ref.
Acticoat^TM^, Smith and Nephew	A low adhesive wound dressing material with a rayon–polyester core meshed into two layers of high density polyethylene, coated with nanocrystalline silver.	Rapid and controlled release of Ag into the wound within a short period of time, leading to enhanced antibacterial activity. The rayon–polyester core absorbs and maintains the moisture, providing the wound and dressing surface with a moist environment. Inhibits growth of >150 microorganisms, including resistant strains.	Moisture from water is needed to activate the dressing. Saline should not be used to moisturise the dressing, as it will deactivate the silver.	[40,45,46,47]
Aquacel^TM^ Ag, ConvaTec	An absorbent non-woven pad or ribbon dressing made of a hydrofibre inlaid with ionic silver.	Highly absorbent and takes up large amounts of wound exudate, so that the silver can exert its effect on the bacteria in the fluids. It has shown excellent antimicrobial activity, including microorganisms that are resistant to antibiotics. The absorbed exudate forms a gel that intimately conforms to the surface of the wound and maintains moist surroundings, assisting autolytic debridement. The dressing can be used for cavity wounds and is non-adhesive. It can be removed from the wound site without causing pain.	Toxic effects have been previously reported. Using Aquacel^TM^ Ag in clinical trials is quite challenging, as insufficient amounts of wound exudates are often collected from the sample, due to the dressing’s highly absorptive properties.	[40,46,48]
Contreet^TM^, Coloplast	A polyurethane (PU) foam dressing incorporated with an ionic silver complex.	Releases silver into the wound bed when it is in proximity with wound exudate. Silver is released in a steady manner, up to seven days. Has antibacterial activity against wound-associated strains, such as *P*. *aeruginosa, S*. *aureus*, *streptococcus*, MRSA and Vancomycin-resistant Enterococcus.	The Ag in the dressing is released per exudate excretion. This could be beneficial, but in cases of high-exudating wounds, an excessive amount of Ag could be released, which could have toxic effects.	[40,46,49]
Actisorb^TM^ Silver 220, Johnson and Johnson	An activated charcoal cloth impregnated with silver within a spun-bonded nylon envelope.	Eliminates infectious microorganisms from wound fluids and exudates by Ag ions inside the charcoal fibres of the wound dressing. The dressing is highly absorbent and slowly releases the Ag over time. Eliminate bad odour. Used for pressure ulcers and leg ulcers.	Although no discolouration (agyria) has been reported, large amounts of Ag can accumulate in the wound site. Difficult to use in clinical trials, since it absorbs most of the wound exudates and none is left for analysis.	[40,46,50]
Urgotul SSD^TM^, Urgo	A gauze dressing coated in a Vaseline^®^ paste, containing carboxymethylcellulose hydrocolloidal particles and SSD.	Administers SSD into the wound to exert its antimicrobial effects against a broad spectrum of bacteria, moulds and yeasts. Useful for treating superficial or deep second-degree burns, which are prone to infections. The dressing is comfortable, can be used anywhere on the body. It can be easily removed from the wound without causing any pain.	Has a limited absorption capacity. Used for low-exudating wounds. Must be used in conjunction with a secondary absorbent dressing for medium to heavily exuding wound.	[40,46,49,51]
Avance^TM^, Mölnlycke	PU foam dressing containing a silver zirconium phosphate complex.	Provides sufficient moisture and hydration to the wound. The silver complex inactivates microorganisms in the wound exudates and fluids. The outer layer of the dressing is water-resistant and prevents any bacterial contact.	Minimal antibacterial effect compared to Acticoat^TM^ and Contreet-H. Clinical data on the dressing are limited.	[40,47]

## Data Availability

All the information in this manuscript was obtained from published studies and is referenced accordingly.
